# Investigating the adherence factors of *Escherichia coli* at the bovine recto-anal junction

**DOI:** 10.1128/spectrum.01270-24

**Published:** 2024-09-27

**Authors:** Erin M. Nawrocki, Indira T. Kudva, Edward G. Dudley

**Affiliations:** 1 Department of Food Science, The Pennsylvania State University, University Park, Pennsylvania, USA; 2 Food Safety and Enteric Pathogens Research Unit, National Animal Disease Center, Agriculture Research Service, U.S. Department of Agriculture, Ames, Iowa, USA; 3 E. coli Reference Center, The Pennsylvania State University, University Park, Pennsylvania, USA; Indian Institute of Technology Hyderabad, Hyderabad, Telangana, India

**Keywords:** *Escherichia coli*, Shiga toxins, cattle, adherence

## Abstract

Shiga toxin**–**producing *Escherichia coli* (STEC) are major foodborne pathogens that result in thousands of hospitalizations each year in the United States. Cattle, the natural reservoir, harbor STEC asymptomatically at the recto-anal junction (RAJ). The molecular mechanisms that allow STEC and non-STEC *E. coli* to adhere to the RAJ are not fully understood, in part because most adherence studies utilize human cell culture models. To identify a set of bovine-specific *E. coli* adherence factors, we used the primary RAJ squamous epithelial (RSE) cell-adherence assay to coculture RSE cells from healthy Holstein cattle with diverse *E. coli* strains from bovine and nonbovine sources. We hypothesized that a comparative genomic analysis of the strains would reveal factors associated with RSE adherence. After performing adherence assays with historical strains from the *E. coli* Reference Center (*n* = 62) and strains newly isolated from the RAJ (*n* = 15), we used the bioinformatic tool Roary to create a pangenome of this collection. We classified strains as either low or high adherence and using the Scoary program compiled a list of accessory genes correlated with the “high adherence” strains. While none of the correlations were statistically significant, several gene clusters were associated with the high-adherence phenotype, including two that encode uncharacterized proteins. We also demonstrated that non-STEC *E. coli* strains from the RAJ are more adherent than other isolates and can outcompete STEC in coculture with RSEs. Further analysis of adherence-associated gene clusters may lead to an improved understanding of the molecular mechanisms of RSE adherence and may help develop probiotics targeting STEC in cattle.

**IMPORTANCE:**

*E. coli* strains that produce Shiga toxin cause foodborne illness in humans but colonize cattle asymptomatically. The molecular mechanisms that *E. coli* uses to adhere to cattle cells are largely unknown. Various strategies are used to control *E. coli* in livestock and limit the risk of outbreaks. These include vaccinating animals against common *E. coli* strains and supplementing their feed with probiotics to reduce the carriage of pathogens. No strategy is completely effective, and probiotics often fail to colonize the animals. We sought to clarify the genes required for *E. coli* adherence in cattle by quantifying the attachment to bovine cells in a diverse set of bacteria. We also isolated nonpathogenic *E. coli* from healthy cows and showed that a representative isolate could outcompete pathogenic strains in cocultures. We propose that the focused study of these strains and their adherence factors will better inform the design of probiotics and vaccines for livestock.

## INTRODUCTION

Shiga toxin-producing *Escherichia coli* (STEC) are major foodborne pathogens that are estimated to cause thousands of hospitalizations and dozens of deaths each year in the United States (1). The annual costs of these STEC infections exceed $400 million (2). Contaminated meat, produce, and dairy are all carriers for infection, and illness may result from the consumption of very few organisms (3, 4). Symptoms begin with abdominal distress and diarrhea and may progress to hemorrhagic colitis and hemolytic uremic syndrome (HUS), the complications of which include acute renal failure and neurological impairment (5–7). Antibiotics are not recommended for treating STEC because of their DNA-damaging effect, which can activate transcription, excision, and release of the Shiga toxin (Stx) bacteriophage and exacerbate an infection (7–9). Surveillance and detection of STEC in the food supply chain are therefore of paramount importance to public health.

Cattle are the natural reservoir of STEC (10–12) and are colonized at the recto-anal junction (RAJ) (13). In order to reduce bacterial loads post-slaughter, animal carcasses are decontaminated by kill steps such as acid washing (14, 15). These measures, along with proper temperature control by consumers, are largely successful in preventing STEC outbreaks from ground beef products. Unfortunately, recent years have seen an increase in outbreaks caused by other sources such as fresh produce (16–18). Because STEC are shed by cattle into the environment, they persist in manure and water and can contaminate nearby farms (18, 19). Eliminating STEC from non-bovine foods presents an additional challenge because these foods undergo fewer and/or milder post-harvest treatments (20). In all cases, contamination of food products can be reduced by intervening at the direct source, i.e., by reducing STEC carriage in cattle (21).

Relative to those involved in human infection, STEC genes that promote bovine colonization are poorly characterized. For instance, while intimin is a critical virulence factor required for STEC adherence to human HEp-2 cells, it is dispensable for adherence to RSE cells (22). Few studies using RSE cells to model STEC adherence have found roles for the Cah autotransporter (23) and curli fimbriae (24) in certain *E. coli* O157:H7 strains. Factors such as type I fimbriae (25) and certain nonfimbrial adhesins (26) contribute to RSE adherence in a strain-specific fashion. Furthermore, there is known diversity in the adherence patterns of various STEC to RSEs, with so-called “supershedder” strains displaying a strongly adherent phenotype (27, 28). These data suggest that within the broad diversity of STEC genomes, there are uncharacterized genes that promote RSE adherence. To our knowledge, however, there has not been a comprehensive survey of RSE adherence factors in generic bovine-associated *E. coli*.

In this work, we have explored RSE adherence in *E. coli* strains of various pathotypes and serotypes to search for adherence factors in the *E. coli* pangenome. Surveying the species in this way may lead to new candidates for drugs, vaccines, or probiotic interventions that reduce the carriage of STEC in cattle and prevent contamination of the food chain.

## RESULTS

### Bovine *E. coli* strains vary in adherence to RSE cells

**Fig 1:**
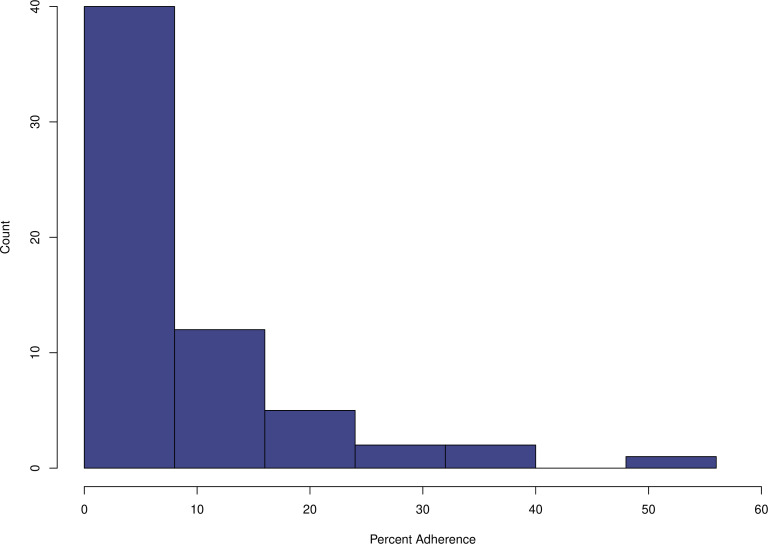
Percent adherence to RSE cells is low. Historical strains with sequenced genomes were selected for adherence assays and pangenome analysis. Of more than 60 ECRC strains tested, most were only weakly adherent. Percent adherence was determined by taking the ratio of CFU/mL RSE-adherent bacteria to CFU/mL total bacteria at the end of 4 h coculture.

**Fig 2:**
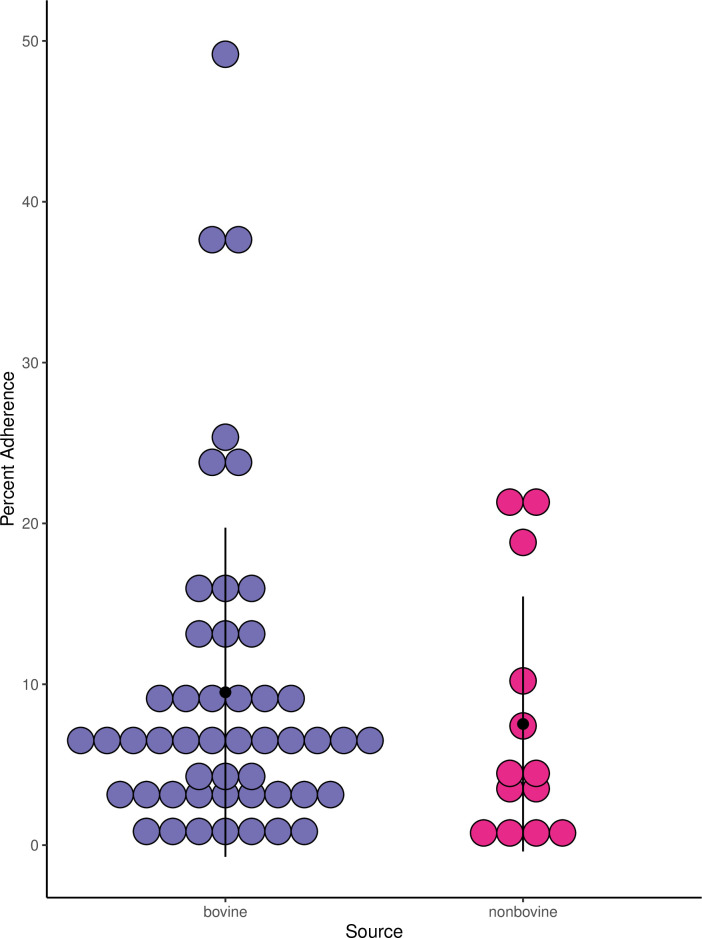
RSE adherence of bovine and nonbovine ECRC isolates is not significantly different. Strains from the ECRC are tagged with metadata including the animal, clinical, or environmental sources from which they were collected. In this sample set, the percent adherence of strains from bovine sources (9.5%, *n* = 49) and the percent adherence of strains from nonbovine sources, including humans and other wild animals (7.5%, *n* = 13), were not statistically different (Welch’s two-sample *t*-test, *P* = 0.46). Percent adherence for each strain is the average of multiple trials.

We first hypothesized that *E. coli* isolated from cattle would be more adherent to bovine RSE cells than those from nonbovine sources. To test this, we chose a set of historical samples from the *E. coli* Reference Center (ECRC) that were diverse in serotype, date of isolation, and geographic source (Table 1). We found that isolates from the ECRC did not display a strongly adherent phenotype when grown in coculture with bovine RSE cells. Most of the 62 strains were less than 10% adherent (Fig. 1). Average adherence to RSE cells was slightly higher in strains from bovine sources (9.5%, *n* = 49) than in strains from nonbovine sources (7.5%, *n* = 13), but the difference was not statistically significant (Fig. 2, Welch’s two-sample *t*-test, *P* = 0.46).

**TABLE 1:** Historical strains used in this study^*a*^

ECRC no.	Location	Source animal	Source tissue	O type	H type	MLST	Phylogroup	Accession no.
11.0312	Tennessee	Cow	Small intestine	55	21	6,572	B2	SAMN05439335
12.2061	Nebraska	Cow	Meat trim	18	14	2,325	A	SAMN05468043
13.1306	New Mexico	Cow	Feces	85	8	6,612	A	SAMN05468041
13.1402	Oklahoma	Cow	Feces	86	27			SAMN04902882
86.0519	Nebraska	Cow	Feces	1	18	1,882	D	SAMN04881308
86.0523	Virginia	Cow	Feces	3	11	10	A	SAMN04881310
86.1149	Iowa	Turkey	Bone marrow	88	18	38	D	SAMN04893484
86.1652	Virginia	Cow	Feces	36	10	6,612	A	SAMN05468068
87.1327	Iowa	Cow	Feces	35	16	6,594	B1	SAMN05604786
87.1422	Iowa	Mink	Feces	124	47	1,146	B1	SAMN05604789
88.0613	Pennsylvania	Cow	Stomach	OX9^*b*^		88	C	SAMN04993019
88.0631	Pennsylvania	Horse	Colon	87	39	10	A	SAMN04993020
88.1285	Nebraska	Pig	Small intestine	112	8	2,532	B1	SAMN04993069
88.1338	Nebraska	Cow	Feces	75	19	88	C	SAMN04993071
88.1452	Arkansas	Cow	Feces	117	11	56	B1	SAMN04993079
90.0019	Pennsylvania	Cow	Small intestine	OX8		162	B1	SAMN04993119
90.1734	India	Cow	Milk	OX8				SAMN04993180
90.1865	Canada	Cow	Feces	32	7			SAMN04992395
90.1899	Canada	Cow	Feces	174	39	10	A	SAMN04992402
76.0065	Pennsylvania	Cow	Intestine	136	40	2,008	A	SAMN04279355
78.0002	Colorado	Chicken	Feces	5	32	603	B1	SAMN04279344
78.0138	Colorado	Cow	Feces	91	10	11,202	A	SAMN04279345
78.0260	Colorado	Pig	Feces	19	16	6,558	C	SAMN04279348
79.0182	Colorado	Cow	Feces	6	39	362	D	SAMN04992265
80.0340	Colorado	Cow	Feces	103	15	86	B1	SAMN04992271
81.0084	Colorado	Human	Feces	130	27			SAMN05452886
81.0479	Colorado	Cow	Feces	15	28	542	A	SAMN04992291
82.0277	Pennsylvania	Cow	Small intestine	109	14	5,236	B1	SAMN04992293
82.0282	Iowa	Cow	Feces	OX28		1,081	B1	SAMN04279551
82.0528	Tennessee	Cow	Small intestine	121	21	10	A	SAMN05440404
83.0174	Pennsylvania	Pig	Intestine	OX5		641	Unknown	SAMN05452877
83.0284	Pennsylvania	Cow	Intestine	162	8	6,517	A	SAMN05452871
84.0468	Pennsylvania	Cow	Small intestine	OX1	21	1,830	A	SAMN05440381
85.0243	Pennsylvania	Cow	Feces	154	25	3,090	B1	SAMN04993219
85.0328	Pennsylvania	Cow	Feces	36	10	31	D	SAMN05452886
85.1091	Pennsylvania	Cow	Feces	OX8	49	109	B1	SAMN05452912
9.0495	California	Cow	Feces	55	36	88	C	SAMN05439348
10.0845	Colorado	Cow	Feces	OX6	8	58	B1	SAMN05439342
10.0846	Colorado	Cow	Feces	73	16	336	B1	SAMN05468015
99.0638	Iowa	Cow	Feces	88	44			SAMN02436554
0.0126	Pennsylvania	Cow	Feces			11,039	B1	SAMN04279359
0.1288	California	Cow	Feces	157	7	1,086	B1	SAMN05439349
1.0984	Michigan	Cow	Rectum	2	42	155	B1	SAMN04279328
72.0058	Pennsylvania	Dog	Feces	96	21	11	E	SAMN08355174
72.0066	Pennsylvania	Cow	Feces	55	9	1,280	F	SAMN04279337
70.0154	Pennsylvania	Sheep	Intestine	174	12			SAMN02436330
70.0165	Pennsylvania	Cow	Feces	71	9	46	A	SAMN08368001
71.0065	Pennsylvania	Cow	Intestine	85	16	155	B1	SAMN08367923
2.3019	New York	Cow	Feces			961	B2	SAMN08368008
2.3105	Pennsylvania	Cow	Feces			453	B1	SAMN04279383
2.3106	Pennsylvania	Dog	Feces					SAMN02436552
3.3433	Connecticut	Cow	Jejunum	23	3	345	B1	SAMN04279445
75.0008	Pennsylvania	Cow	Feces	116	6	154	B1	SAMN04279449
75.0067	Pennsylvania	Pig	Lung	23	15	58	B1	SAMN04279488
75.0217	Pennsylvania	Deer	Intestine	OX9		4,035	B1	SAMN04279488
75.0220	Pennsylvania	Cow	Intestine	45	19	718	B1	SAMN05439472
4.0522	Pennsylvania	Cow	Ileum	111		336	B1	SAMN05439471
5.0588	Pennsylvania	Cow	Small intestine	8		4,035	B1	SAMN05439484
6.0668	Texas	Cow	Feces	8	36	345	B1	SAMN04279445
6.1088	Minnesota	Cow	Feces	86	11	1,434	A	SAMN05604798
7.3207	New York	Cow	Uterus	58	27	2,522	B1	SAMN04902878
8.0055	California	Cow	Feces	2	27	101	B1	SAMN04902882

^*a*^ The year of isolation is denoted by the numbers preceding the decimal. O and H types were determined at the ECRC using standard antisera.

^*b*^ “OX” antigens were untypeable using traditional *E. coli* antisera (29, 30). Missing MLSTs and phylogroups are due to incomplete assembly records at NCBI and/or Enterobase.

### RAJ-associated *E. coli* encode various adherence factors

We next sequenced genomes of RAJ isolates and evaluated their adherence to RSE cells. In total, we sequenced 33 presumptive *E. coli* isolated from the RAJ of healthy cattle and confirmed the species assignment for all but one (Table 2). The remaining isolate, 23.0037, was identified as *E. fergusonii* (Table 2). The *E. coli* RAJ isolates each belonged to either phylogroup A or phylogroup B1 (Table 2). In fact, we found that many strains differed by only a few SNPs, suggesting that certain lineages are predominant at the RAJ or, more likely, were overrepresented in our sampling. In accordance with animal protocols, we only swabbed three cows per collection date, and all subjects were housed in the same research facility. For strains that belonged to the same SNP cluster in the Pathogen Detection database (i.e., those that were within five SNPs of one another), we chose one strain from the cluster at random and excluded the others from our adherence and virulence factor analyses, leaving 15 non-clonal RAJ strains for genetic comparisons.

**TABLE 2:** Sequencing statistics for RAJ-associated *E. coli* strains^*a*^

ECRC no.	N50	Length	Contigs	Predicted serotype	MLST	Phylogroup	Accession no.
23.0001	221,856	4,921,956	100	O102:H21	155	B1	SAMN36717240
23.0004	96,900	5,235,930	237	O4:H37	10	A	SAMN36717241
23.0006	55,271	4,839,925	220	O89:H38	154	B1	SAMN36717242
23.0007	77,549	4,869,276	157	O89:H38	154	B1	SAMN36717243
23.0008	135,155	4,906,030	95	O89:H38	154	B1	SAMN36717244
23.0009	125,242	4,816,384	113	O89:H38	154	B1	SAMN36717245
23.0010	125,242	4,916,631	105	O89:H38	154	B1	SAMN36717246
23.0011	98,504	5,597,033	266	-:H7	1,101	A	SAMN36717247
23.0012	60,477	5,625,648	291	-:H7	1,101	A	SAMN36848571
23.0013	79,461	5,652,830	275	O39:H7	1,101	A	SAMN36717248
23.0015	101,577	5,614,057	246	O39:H7	1,101	A	SAMN36717250
23.0018	66,884	5,614,385	292	O39:H7	1,101	A	SAMN36717251
23.0019	353,635	4,776,404	67	O104:H23	154	A	SAMN36717252
23.0020	72,289	4,701,900	164	O155:H34	1,415	A	SAMN36717253
23.0021	103,578	4,782,492	116	O104:H23	939	A	SAMN36848572
23.0023	66,900	4,689,973	167	O155:H34	1,415	A	SAMN36848573
23.0024	74,085	4,779,155	179	O8:H54	58	B1	SAMN36717254
23.0026	209,663	4,897,698	69	-:H21	1,248	B1	SAMN36717255
23.0027	66,900	4,906,518	195	:H12	1,326	B1	SAMN36717256
23.0028	211,124	4,842,290	61	O128:H12	1,326	B1	SAMN36717257
23.0030	126,818	4,894,406	112	:H21	1,248	B1	SAMN36717258
23.0031	154,257	4,908,410	101	:H21	1,248	B1	SAMN36848574
23.0032	136,708	4,904,705	110	:H21	1,248	B1	SAMN36848575
23.0033	192,949	4,902,149	78	:H21	1,248	B1	SAMN36717259
23.0034	270,014	4,748,484	53	O113:H7	196	B1	SAMN36717260
23.0035	151,765	4,890,898	76	O88:H25	154	B1	SAMN36717261
23.0036	151,938	4,860,395	83	O88:H25	154	B1	SAMN36717262
23.0037	60,810	4,796,486	152	N/A	316	N/A	SAMN36717263
23.0038	57,889	4,827,967	213	O88:H25	154	B1	SAMN36717264
23.0049	118,803	5,070,777	124	O162/O101:H7	316	B1	SAMN36717265
23.0050	147,062	4,994,566	125	O2/O50:H42	164	B1	SAMN36848576
23.0051	295,895	4,981,446	52	:H21	1,248	B1	SAMN36717266
23.0052	125,487	5,062,446	121	O162/O101:H7	316	B1	SAMN36848577

^*a*^ All were isolated from cows housed at Penn State’s bovine research facilities. They were sequenced using paired-end reads on an Illumina MiSeq and are deposited in the NCBI under BioProject PRJNA357722.

**Fig 3:**
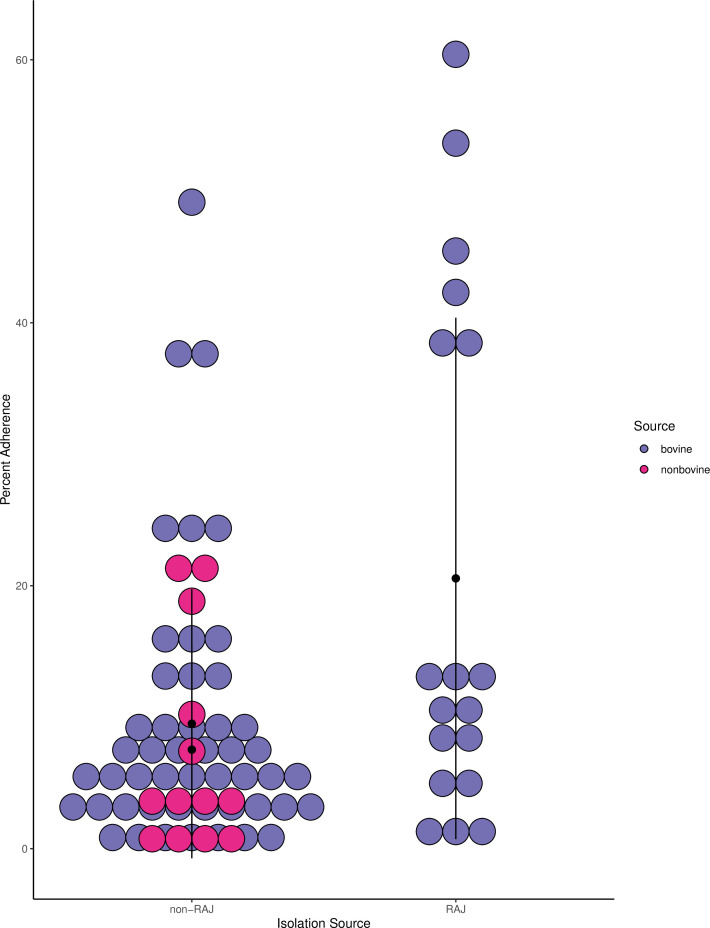
RAJ strains are more adherent than non-RAJ strains. Percent adherence of historical strains from the ECRC (Fig. 2) was compared to that of newly isolated RAJ strains. Those from the RAJ displayed a higher percent adherence (20%, *n* = 15) than those from other sources (9%, *n* = 62). The difference was significant according to Welch’s two-sample *t*-test, *P* = 0.03.

When RSE adherence assays were repeated with strains newly isolated from the RAJ, adherence values were notably increased. The fifteen RAJ strains averaged 20% adherence to RSEs, which was significantly higher than the 9% average adherence of the sixty-two non-RAJ strains (*P* = 0.03; Fig. 3; Table S1).

**Fig 4:**
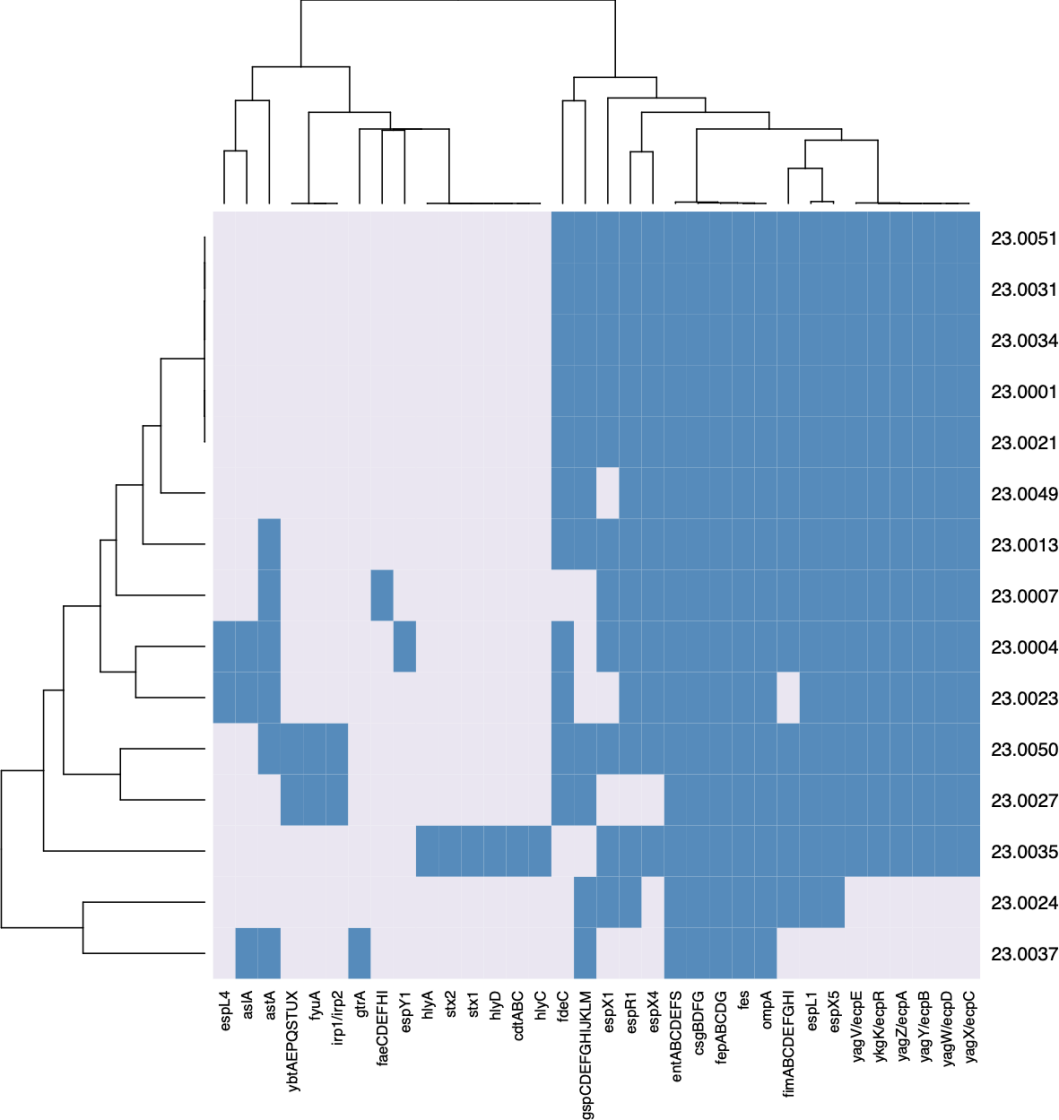
Virulence-associated genes are rare in RAJ isolates. Genome sequences were generated on the Illumina MiSeq platform and submitted to NCBI’s Pathogen Detection database. Virulence genes were identified using the VFDB and visualized using RStudio.

Analysis of the RAJ genomes for virulence factors indicated that several gene families were conserved in this group (Fig. 4). The *entABCDEFS*, *csgBDFG*, *fepABCDG*, *fes*, and *ompA* genes were all present in 100% (15/15) of isolates (Fig. 4). Other common virulence-associated genes in the RAJ strains included *espL1* and *espX5* (each 93%, 14/15); *fimABCDEFGHI* (87%, 13/15); and *ecpABCDER*/*yagZYXWVK* (87%, 13/15). Toxin genes were rare, although a subset of the strains carried either *astA*, a heat-stable enterotoxin (40%, 6/15), or *stx1* and *stx2*, the two types of Shiga toxin (7%, 1/15).

### Bovine *E. coli* strains have a large accessory genome

**Fig 5:**
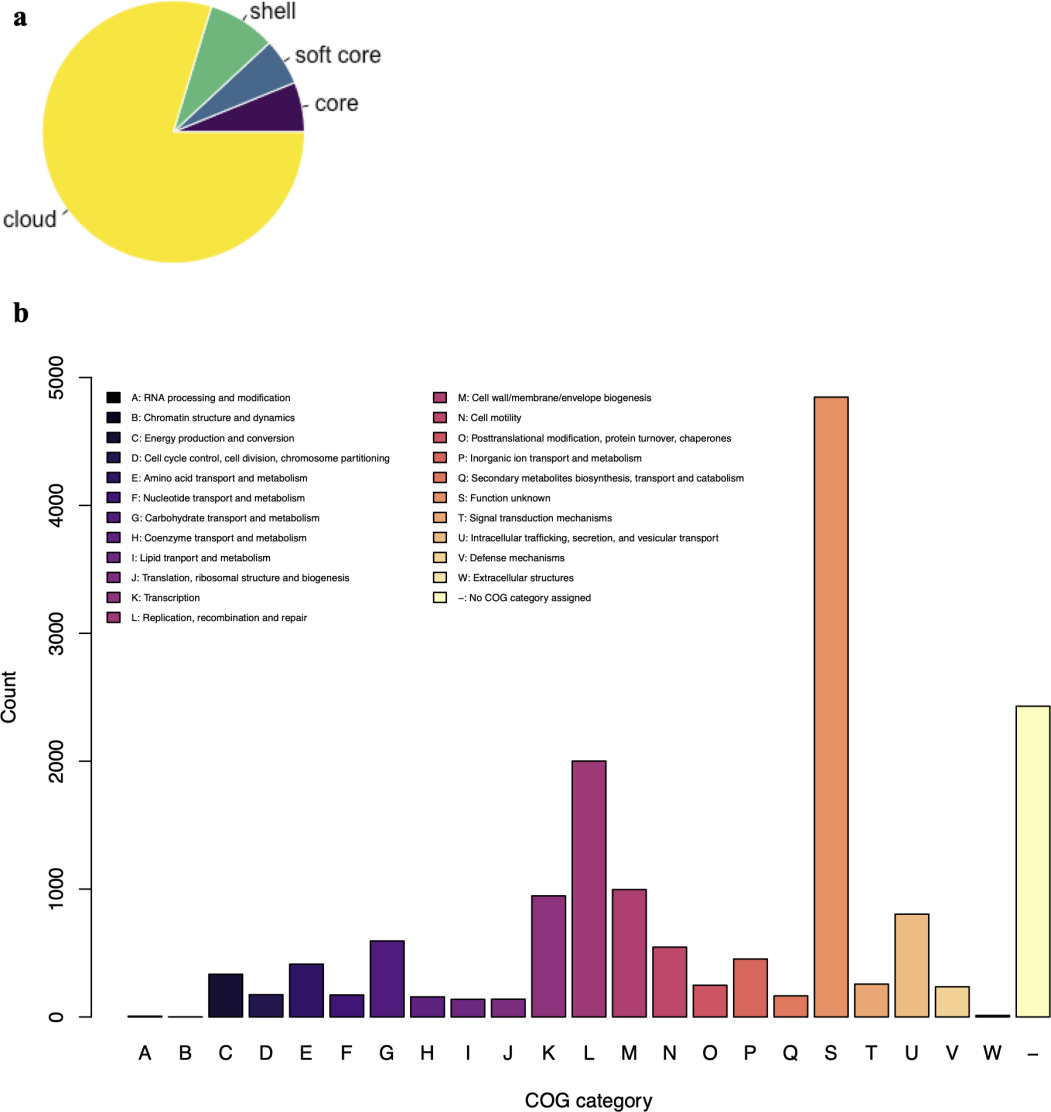
Most accessory gene clusters in the pangenome are functionally uncharacterized. (a) Roary analysis of ECRC strains revealed a small core genome (3,117 core + soft core gene clusters, each present in > 95% of strains) and a large accessory genome (23,089 shell + cloud gene clusters). (b) Unique cloud, shell, and soft-core genes were collected into a single FASTA file and annotated at the eggNOG-mapper webserver. The output was downloaded in tabular format and parsed and plotted with a custom R script. Note that some genes are assigned to more than one COG category.

While some of the operons above encode adhesion-related molecules, such as *csg* (curli), *fim* (type I fimbriae), and *ecp* (*E. coli* common pilus), they are likely not responsible for the improved adherence we observed in RAJ strains—these genes are each encoded by the majority of ECRC isolates, including those with low adherence. To expand our search for adherence factors, we generated a pangenome of the full strain set (ECRC strains and RAJ strains combined) so that we could evaluate all possible accessory gene clusters for associations with RSE adherence. According to analysis performed using Roary, the full set of strains contains 3,117 gene clusters that are part of the core or soft-core genome, that is, are present in >95% of strains. The remaining 23,089 gene clusters comprise the accessory genome of these strains (Fig. 5a). Most of the accessory gene clusters either do not have a Clusters of Orthologous Genes (COG) category assigned or are classified as category S, “function unknown” (Fig. 5b).

To determine which gene clusters were associated with RSE adherence, we binned each strain as either low or high adherence using a breakpoint of 8%. Analysis with Scoary revealed three genes that were most significantly associated with the high-adherent phenotype: *marB*, *yneK*, and *yncI* (naïve *P*-values < 0.005, best and worst pairwise comparison *P*-values < 0.05). *marB* (sensitivity 100%; specificity 33%) is predicted to encode a multiple antibiotic resistance protein. *yneK* (sensitivity 95%; specificity 42%) and *yncI* (sensitivity 81%; specificity 61%) are both annotated as putative proteins. Various ORFs with adherence-related annotations are also among the Scoary results, including a putative pilin chaperone *yadV* and putative fimbrial-like adhesins *yadM*, *yadN*, and *yfcV*. A full list of adherence-associated genes is provided in Table S2. When adjusted for multiple comparisons, all genes had *P*-values equal to 1, indicating that our GWAS was not sufficient to detect associations between genotypic and phenotypic adherence at this scale (Table S2).

### RAJ *E. coli* can outcompete EHEC in coculture

**Fig 6:**
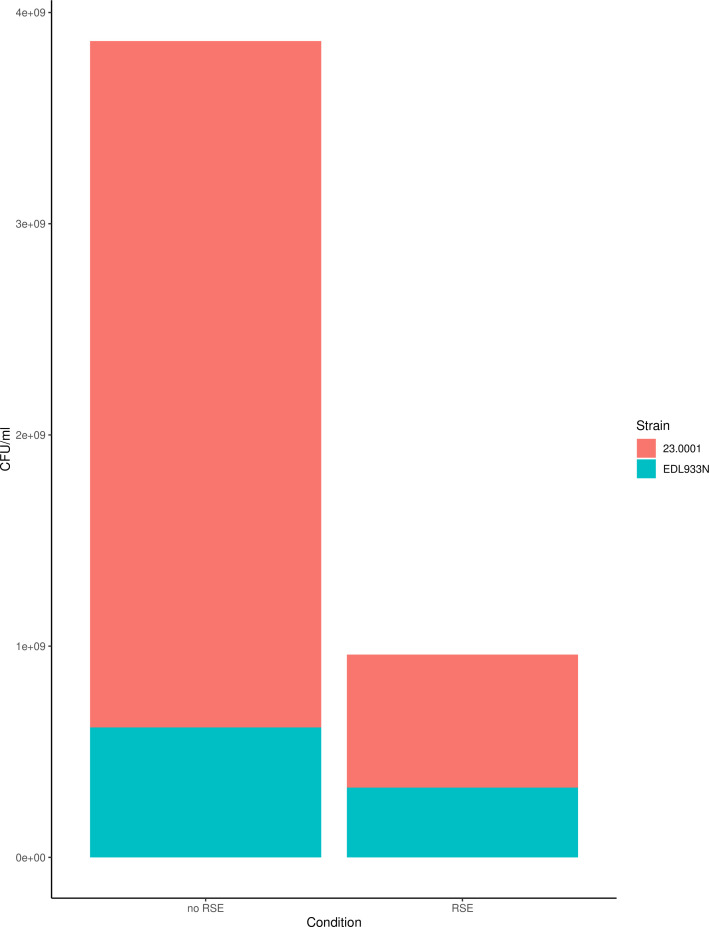
The RAJ strain 23.0001 outcompetes a representative EHEC strain. Strains were mixed at equal cell densities and grown for 24 hours in DMEM-LG. The RAJ-associated *E. coli* grow better and were more adherent to RSE cells than their *E. coli* O157:H7 counterparts. The mean CFU/mL for each condition (*n* = 6 without RSE; *n* = 2 with RSE) is reported. RAJ strain 23.0001 was significantly more fit than EHEC strain EDL933N when grown in coculture without RSE cells (*P* = 0.03) and outcompeted EDL933N when grown in coculture with RSE cells, although the difference was not statistically significant at this sample size (*P* = 0.1, Welch’s two-sample *t*-test).

Lastly, seeing that RAJ strains successfully adhered to RSE cells, we asked whether they were fitter than EHEC when grown in coculture with RSEs, as this would be an asset for strains used as direct-fed microbials. Strain 23.0001 was chosen for this experiment due to its inherent tetracycline resistance, which facilitated selection from coculture. When mixed at equal proportions and grown in DMEM-LG without antibiotics for 24 hours, 23.0001 maintained a significant competitive advantage over EDL933N, a spontaneous Nal^R^ mutant of the O157:H7 strain (*P* = 0.03, Welch’s two sample *t*-test). When the two were mixed with RSE cells in suspension coculture, 23.0001 again outcompeted the pathogenic strain, although the difference was not as substantial (Fig. 6).

## DISCUSSION

In this work, we sought to identify bovine-specific adherence factors in *E. coli* using two complementary approaches. First, we performed a genome-wide association study using historical strains from the *E. coli* Reference Center. Second, we isolated new *E. coli* strains from the RAJ of healthy cattle, assessed their adherence, and searched their genomes for specific virulence factors. Although the average adherence of ECRC strains was low, we found several gene clusters in our sample set that were weakly associated with RAJ colonization. In addition, we observed significantly higher adherence in RAJ strains than in historical isolates, and we suggest that RAJ *E. coli* be further investigated for probiotic approaches to STEC control and prevention.

Because we hypothesized that ECRC strains isolated from cattle would display increased adherence in coculture with cattle cells, we initially chose sequenced strains from the ECRC whose collection source was given as “cow” or “bovine.” In fact, we found that the adherence of bovine and non-bovine ECRC isolates did not differ. Imprecision in the metadata could explain this result. Bovine *E. coli* do not constitute a true pathotype, and ECRC strains from bovine sources may have been collected from any tissue or organ in the animal. For instance, some strains were isolated from milk or reproductive organs, not from the colon (Table 1). Even those that are from the gastrointestinal tract may not have specific tropism for the RAJ, which is enriched in epithelial cells and lymphoid follicles at only the most distal part of the colon (13). Underscoring this point, we performed an association study in Scoary comparing the pangenomes of bovine and non-bovine strains, and we did not find any significant (naïve *P*-values < 0.005, best and worst pairwise comparison *P*-values < 0.05) gene clusters that we considered markers of bovine strains (Table S3).

Closer examination of the gene clusters of *marB*, *yneK*, and *yncI* does not clarify their role in adherence to RSE cells. MarB is a small periplasmic protein with roles in regulating multidrug resistance (31). It is thought to interact with cytoplasmic transcription factors only indirectly, and thus it is not known whether it modulates an adherence factor. YneK is uncharacterized, and its only conserved domains are disordered regions (UniProt: P76150). Interestingly, the genes encoding *marB* and *yneK* are only 4.5 kb apart on the *E. coli* MG1655 chromosome, which raises the possibility that they are linked to an unknown effector and do not contribute directly to adherence. While *marB* and *yneK* are both intact on the nonpathogenic *E. coli* MG1655 chromosome, *yncI* in MG1655 is a pseudogene containing a premature stop codon. In the STEC O157:H7 strain Sakai, however, *yncI* is present in full. This SNP difference could indicate a functional role for *yncI*—which is annotated as an ISEc26 family transposase—in the pathogen. Further investigation of the molecular mechanisms of these gene products is necessary to confirm their role in adherence.

The observation that RAJ strains are more adherent than the broader bovine set of ECRC strains is not surprising. When comparing various serotypes of bovine commensal *E. coli*, Naylor et al. found that they were not uniformly distributed in the gastrointestinal tract and that STEC *E. coli* O157:H7 were concentrated in the terminal rectum (13). Although none of our RAJ strains were serotype O157:H7 (Table 2), they were also isolated from the terminal rectum. The presence of *E. coli* at this site is associated with high fecal counts and longer fecal shedding, which suggests that colonization at the RAJ is persistent and stable (32, 33). Our RSE adherence assays recapitulated this *in vitro*, albeit on a much shorter timescale. Additional research into the long-term population structure and stability of RAJ strains, including testing their adherence to more complex tissue substrates like the RAJ-IVOC model (34), may help support their use as direct-fed microbials (DFMs). Although DFMs are an attractive strategy for addressing STEC contamination in cattle, they have often been unsuccessful in colonizing the lower gastrointestinal tract (35, 36). A longitudinal study to isolate and sequence additional RAJ strains over time could reveal traits associated with persistence and would contribute to our understanding of adherence factors in *E. coli*.

Finally, it is possible that the Roary and Scoary analyses are insufficient to identify bona fide adherence genes in our *E. coli* sample set. These tools are based on the presence and absence of genes, categories which may be too crude to recognize certain adherence factors. A core gene that is present in all strains but has different variants or regulatory elements would not yield a significant Scoary association with adherence. For instance, two of the ECRC strains with the highest percent adherence encode the *lpfA*-O113 adhesin, an allele of the conserved *lpfA* gene cluster that is involved in adherence to porcine cells (37). To address the role of core and conserved genes, a more powerful genome-wide association analysis would ideally incorporate more *E. coli* strains and a more precise (e.g., SNP- or k-mer-based) variant caller. In addition, we suggest that subsequent research into RSE cell adherence might explore transcriptional activity, in both the bacteria and the bovine cells, to contextualize the relationship between the two.

## MATERIALS AND METHODS

### Bacterial strains and culture conditions

Forty-nine bovine and 13 non-bovine *E. coli* strains were selected from Penn State’s *E. coli* Reference Center (ECRC) based on available metadata and complete whole-genome sequences (WGS). Strains were chosen at random, with an effort to include various serotypes, geographical origins, and isolation years. WGS data for these strains are publicly available at the NCBI’s Pathogen Detection site (https://www.ncbi.nlm.nih.gov/pathogens) under the accession numbers in Table 1. Routine growth and proliferation of *E. coli* were accomplished in LB media at 37°C. Antibiotics were used at the following concentrations: ampicillin, 50 µg/mL; chloramphenicol, 12.5 µg/mL; kanamycin, 25 µg/mL; nalidixic acid, 30 µg/mL; streptomycin, 100 µg/mL; and tetracycline, 10 µg/mL. Each bacterial isolate was revived from frozen stock (LB supplemented with 20% glycerol) and screened for growth on LB plates containing each of the antibiotics above. If an isolate was sensitive to all antibiotics, a colony was grown in 3 mL LB overnight, pelleted by centrifugation, resuspended in 100 µL LB, and plated on LB agar with nalidixic acid. Plates were incubated overnight to allow for the growth of spontaneous Nal^R^ mutants. Nal^R^ mutant derivatives were used in place of the pan-susceptible parent strains to facilitate recovery from cocultures. Coculture assays were performed in Dulbecco’s modified Eagle medium with 0.1% glucose (DMEM-LG). All chemicals were purchased from Life Technologies or VWR Scientific unless otherwise noted.

### Isolation of RSE cells

RSE cells were isolated from healthy Holstein cattle at Penn State’s Mastitis and Dairy Barns. A recto-anal mucosal swab (RAMS) technique (38) was used to collect cells from each animal: a foam-tipped applicator was inserted 2–5 cm into the anus and rapidly swabbed over the entire surface. The swab was deposited into 10 mL DMEM without glucose, supplemented with 2.5% fetal bovine serum, 100 µg/mL streptomycin, 100 U/mL penicillin, and 50 µg/mL gentamicin (DMEM+NG), and kept on ice. This process was repeated five additional times for a total of six RAMS samples per animal. Swabs were collected from three different animals on each sampling date. The procedures described were approved and authorized by Penn State’s Institutional Animal Care and Use Committee under protocol number PROTO202101907.

Swabs in DMEM+NG were transported back to the laboratory and processed as previously described (39). In summary, the RAMS samples from each animal were pooled, filtered through a 70-µm cell strainer, and centrifuged at 850 x *g* at 4°C for 15 minutes. This process was repeated twice to remove as much fecal debris from the cells as possible. An aliquot of the remaining pellet was removed and stained with 0.4% trypan blue to count squamous epithelial cells, and the cells were suspended in 50% glycerol at 10^5^ cells/mL for long-term storage at −80°C.

### Adherence assays

RSE adherence experiments were performed in suspension cocultures as in prior literature (39). For every coculture assay, *E. coli* were grown overnight in LB and subcultured into DMEM-LG until the culture reached the mid-logarithmic phase. To prepare RSE cells, an aliquot was thawed from frozen stock, suspended in 10 mL DMEM-LG, filtered through a 70-µm nylon cell strainer, and centrifuged for 5 minutes at 850 x *g*. The pellet was suspended in DMEM-LG to a concentration of 10^5^ cells/mL, stained with trypan blue, and counted using a hemocytometer to confirm. One microliter of the log-phase *E. coli* culture (approximately 10^6^ CFU) was then inoculated into 1 mL DMEM-LG containing 10^5^ RSE cells. Controls without *E. coli* and without RSEs were inoculated in parallel. All tubes were gently shaken at 110 rpm at 37°C for 4 hours.

At the conclusion of the assay, an aliquot was removed from each tube and treated with 0.1% Triton X-100 for 5 minutes. This sample was then vortexed, serially diluted, and plated on selective agar to enumerate total *E. coli* in the suspension. The remaining volume was centrifuged for 5 minutes at 300 x *g*, washed with 1 mL DMEM-NG, and washed twice more with 1 mL ultrapure water, pelleting RSE cells and their attached *E. coli*. The final pellet was suspended in 1 mL ultrapure water, treated with 0.1% Triton X-100 to release adhered bacteria, and serially diluted to quantify adherent *E. coli*. The percent adherence of each strain was calculated by taking the ratio of adherent CFU/mL to total CFU/mL. The RSE-only control was plated on selective agar to confirm that there were no contaminating microorganisms in the cell suspension. Total bacteria from the *E. coli*–only control were enumerated as described above and compared to the total CFU/mL from cocultures to demonstrate that RSE cells were not inhibitory to bacterial growth.

### Whole-genome sequencing

RAJ-associated *E. coli* were isolated concurrently with RSE cells. For each animal sampled, two additional RAMS were obtained and transported back to the laboratory in DMEM+NG without antibiotics. Swabs were either directly inoculated onto MacConkey agar (MAC) or enriched in transport medium overnight at 37°C before selection on MAC. Lactose-fermenting colonies were confirmed as *E. coli* by production of indole, failure to utilize citrate, and PCR amplification of the *uidA* gene (40).

A single colony of each isolate was grown aerobically in LB (37°C, 220 rpm) for 16–18 hours. Genomic DNA from this culture was extracted with the Qiagen DNeasy Blood and Tissue kit and eluted in nuclease-free water. Samples were barcoded and indexed for Illumina sequencing using the Nextera XT Library Preparation Kit. Whole-genome sequencing was performed on Illumina’s MiSeq platform employing v3 chemistry and 2 × 250-bp paired-end reads. FASTQ files were checked for quality and assembled into contigs using the MicroRunQC_v1.2 workflow available in GalaxyTrakr. Contigs were screened for virulence genes in GalaxyTrakr using ABRicate against the Virulence Factor Database (VFDB) (41, 42). Reads were uploaded to NCBI’s Pathogen Detection database (https://www.ncbi.nlm.nih.gov/pathogens; accession numbers in Table 2), where they were reassembled and joined into SNP clusters (43–45). An SNP phylogeny of all RAJ isolates is available as Fig. S1. Sequence types and phylogroups were obtained from the records of assembled genomes in EnteroBase (46).

### Bioinformatic analysis

Genome assemblies of the historical ECRC strains were downloaded in FASTA format from the NCBI. All sequenced ECRC strains are available under BioProject accession number PRJNA357722 and/or the BioSample number in Table 1. Each assembly was locally re-annotated using Prokka (47) for uniformity. The Prokka outputs were used to generate a pangenome using Roary (4). All parameters were unchanged from default settings. Strains were binned into two groups according to their adherence to RSE cells, where adherence less than 8% was categorized as “low” and adherence greater than or equal to 8% categorized as “high.” Low and high adherence were represented as 0 and 1, respectively, and used as inputs for trait association analysis by Scoary (48). Heatmaps of virulence genes were plotted in R version 3.6.3 using the *stats* and *RColorBrewer* 1.1–2 packages (49, 50).

## SUPPLEMENTAL MATERIAL

Figure S1 spectrum.01270-24-s0001.pdf SNP phylogeny of RAJ isolates.

Table S1 spectrum.01270-24-s0002.csv RSE adherence assay data.

Table S2 spectrum.01270-24-s0003.csv Scoary adherence-associated genes.

Table S3 spectrum.01270-24-s0004.csv Scoary bovine-associated genes.

ASM does not own the copyrights to Supplemental Material that may be linked to, or accessed through, an article. The authors have granted ASM a non-exclusive, world-wide license to publish the Supplemental Material files. Please contact the corresponding author directly for reuse.
