# Supplementary material for: Investigating the adherence factors of Escherichia coli at the bovine recto-anal junction
Source: Microbiol Spectr. 2024 Sep 27;12(11):e01270-24. doi: 10.1128/spectrum.01270-24 (PMC11540155; doi:10.1128/spectrum.01270-24)
Supplement: Figure S1 — SNP phylogeny of RAJ isolates. [file spectrum.01270-24-s0001.pdf]

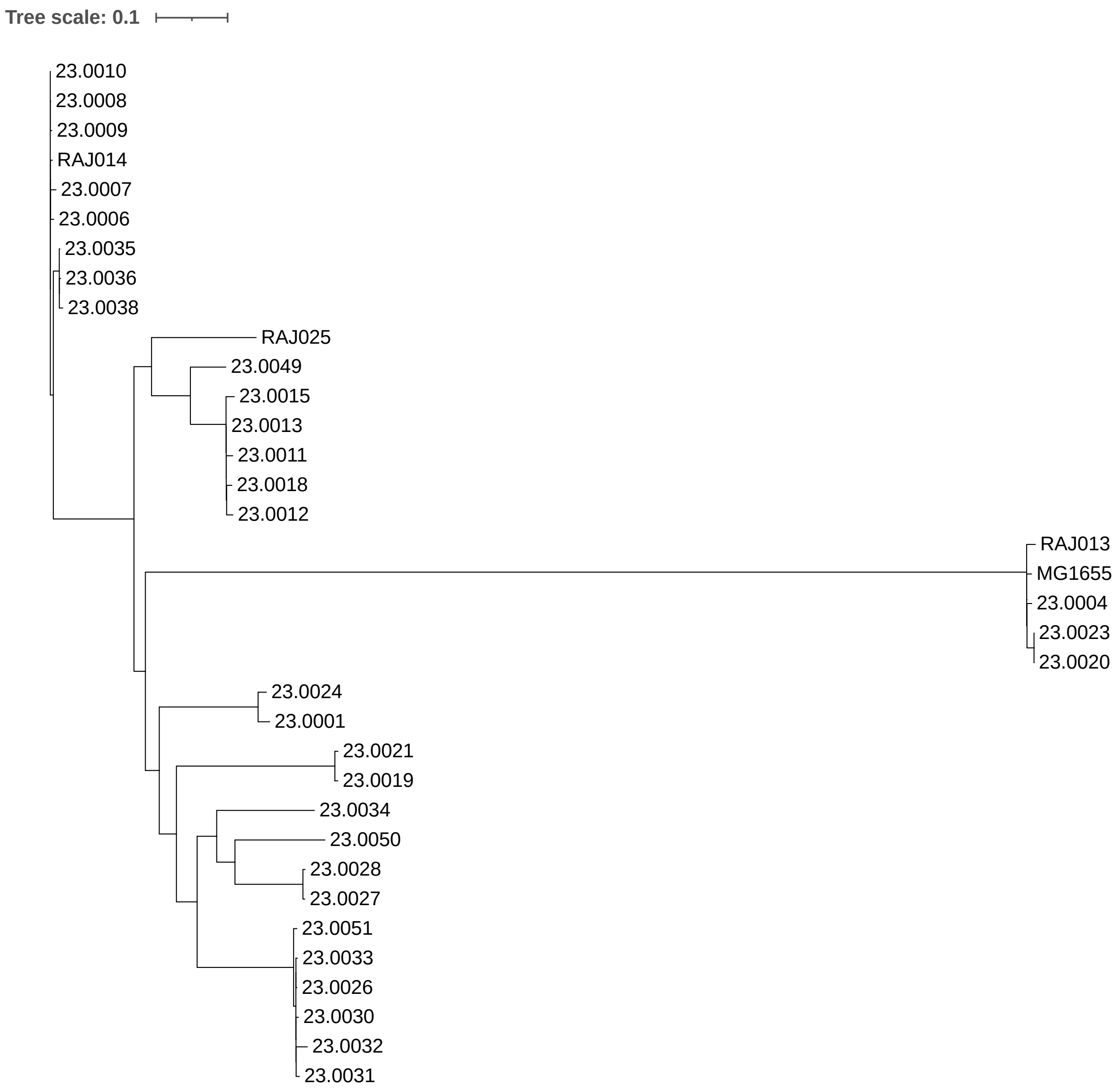

Supplemental Figure 1. SNP phylogeny of *E. coli* strains isolated from the RAJ. Strains were obtained and sequenced as described in the text. Bioinformatic analysis was performed at the GalaxyTrakr webserver: Snippy was used to call and align SNPs in the core genome and FASTTREE was used to generate a phylogenetic tree, which was visualized with iTOL (https://itol.embl.de/).
